# The Impact of Vitamin D Levels on Inflammatory Status: A Systematic Review of Immune Cell Studies

**DOI:** 10.1371/journal.pone.0141770

**Published:** 2015-11-03

**Authors:** Emily K. Calton, Kevin N. Keane, Philip Newsholme, Mario J. Soares

**Affiliations:** 1 Directorate of Nutrition Dietetics & Food Technology, School of Public Health, CHIRI-Metabolic Health, Curtin University, Perth, Western Australia 6845; 2 School of Biomedical Sciences, CHIRI Biosciences, Curtin University, Perth, Western Australia 6845; University of Washington, UNITED STATES

## Abstract

Chronic low-grade inflammation accompanies obesity and its related chronic conditions. Both peripheral blood mononuclear cells (PBMCs) and cell lines have been used to study whether vitamin D has immune modulating effects; however, to date a detailed systematic review describing the published evidence has not been completed. We therefore conducted a systematic review on the effect of vitamin D on the protein expression and secretion of inflammatory markers by human-derived immune cells. The review was registered at the International Prospective Register for Systematic Reviews (PROSPERO, Registration number CRD42015023222). A literature search was conducted using Pubmed, Science Direct, Scopus, Web of Science and Medline. The search strategy used the following search terms: Vitamin D or cholecalciferol or 1,25-dihydroxyvitamin or 25-hydroxy-Vitamin D and Inflam* or cytokine* and supplement* or cell*. These terms were searched in the abstract, title and keywords. Inclusion criteria for study selection consisted of human-derived immune cell lines or cellular studies where PBMCs were obtained from humans, reported in the English language, and within the time period of 2000 to 2015. The selection protocol was mapped according to PRISMA guidelines. Twenty three studies (7 cell line and 16 PBMCs studies) met our criteria. All studies selected except one used the active metabolite 1,25(OH)_2_, with one study using cholecalciferol and two studies also using 25(OH)D. Four out of seven cell line studies showed an anti-inflammatory effect where suppression of key markers such as macrophage chemotactic protein 1, interleukin 6 and interleukin 8 were observed. Fourteen of sixteen PBMC studies also showed a similar anti-inflammatory effect based on common inflammatory endpoints. Mechanisms for such effects included decreased protein expression of toll-like receptor-2 and toll-like receptor-4; lower levels of phosphorylated p38 and p42/42; reduced expression of phosphorylated signal transducer and activator of transcription 5 and decreased reactive oxygen species. This review demonstrates that an anti-inflammatory effect of vitamin D is a consistent observation in studies of cell lines and human derived PBMCs.

## Introduction

Inflammation is recognised as the underlying characteristic of obesity and related chronic disease including type two diabetes [1–3] and cardiovascular disease [4–7]. In fact, inflammation may contribute to a multitude of chronic diseases [8]. Peripheral blood mononuclear cells (PBMCs) play a key role in the development and progression of obesity-related chronic diseases and have recently been suggested to be of potential use as biomarkers of health status [9–11]. Systemic inflammation is characterised by elevated levels of inflammatory biomarkers in the blood stream such as tumour necrosis factor α (TNF-α), interleukin-1β (IL-1β), interleukin-2 (IL-2), interleukin-6 (IL-6), interleukin-8 (IL-8) and interleukin-12 (IL-12).

Inadequate vitamin D status is common in many parts of the world [12] and is associated with obesity and related chronic disease [13–16]. The main source of vitamin D is through endogenous production, whereby solar UV-B irradiates 7-dehydrocholesterol present in the skin to generate cholecalciferol [17, 18], which is subsequently activated in the liver and kidney. The second source of vitamin D is dietary intake, which includes supplementation with either ergocalciferol (vitamin D_2_) or cholecalciferol (vitamin D_3_) [18]. Classifying vitamin D status is based upon the serum levels of 25(OH)D [17, 19]. However, the appropriate level of circulating 25(OH)D required for good health, is hotly debated [20, 21]. Vitamin D is argued by many to have potential extra-skeletal health effects, impacting energy balance and possibly reducing inflammation [21–25]. However, the findings are inconsistent from cross-sectional studies, and human clinical trials that have investigated the potential links between vitamin D status and systemic inflammatory markers [26–29].

Cellular studies indicate that vitamin D is a key modulator of immune function and inflammation [30, 31]. There is an increasing appreciation that vitamin D exerts broad regulatory effects on cells of the adaptive and innate immune system [32]. Current evidence suggests that the circulating level of 25(OH)D may be crucial for the optimal anti-inflammatory response of human monocytes [22]. The conversion of 25(OH)D to its active form 1,25(OH)_2_ occurs locally in immune system cells. The active metabolite of vitamin D has an anti-inflammatory effect on the inflammatory profile of monocytes [17, 33, 34], down-regulating the expression and production of several pro-inflammatory cytokines including TNF- α, IL-1β, IL-6, and IL-8 [33, 34]. Some immediate vitamin D action occurs in cells that possess the membrane vitamin D receptor (mVDR) [35]. However, the majority of vitamin D’s biological functions are mediated through the regulation of gene expression. The active metabolite of vitamin D 1,25 dihydroxyvitamin D (1,25(OH)2D3) binds to its nuclear receptor (nVDR) with high affinity and specificity. The vitamin D-nVDR forms a heterodimer with the retinoid X receptor and this complex amplifies or represses transcription of the target genes through its binding to vitamin D responsive elements on DNA [17]. The nVDR is found in multiple cells of the immune system such as human Treg cells [36], neutrophils [37], dendritic cells, B cells [38] and macrophages [39].

To the best of our knowledge, there are no previously published systematic reviews that comprehensively assess the evidence for anti-inflammatory effects of vitamin D in human derived immune cells and human cell lines. We therefore embarked on this objective by targeting human-derived immune cell lines or PBMCs obtained from healthy participants or those with obesity-related chronic disease. In addition, we aimed to identify the pathways by which vitamin D modulated inflammation. We conclude that vitamin D has an anti-inflammatory effect with respect to cytokine expression and production, in both immune cell lines and PBMCs originating from humans. Furthermore, our review also highlights several mechanisms of action that may explain this anti-inflammatory effect of vitamin D.

## Materials and Methods

This systematic review assessed the effect of vitamin D on the inflammatory profile of immune cells, using both human-derived immune cell lines and PBMCs obtained from adult humans. The primary outcomes were protein expression and secretion of common inflammatory markers such as pro-inflammatory cytokines MCP-1, IL-1β, IL-2, IL-6, IL-8, IL-12, TNF-α, CRP and anti-inflammatory markers such as IL-10 and IL-4 by immune cells. The protocol has been registered at the International Prospective Register for Systematic Reviews (PROSPERO) website (registration number CRD42015023222, S1 Table. Systematic review protocol).

**Fig 1:**
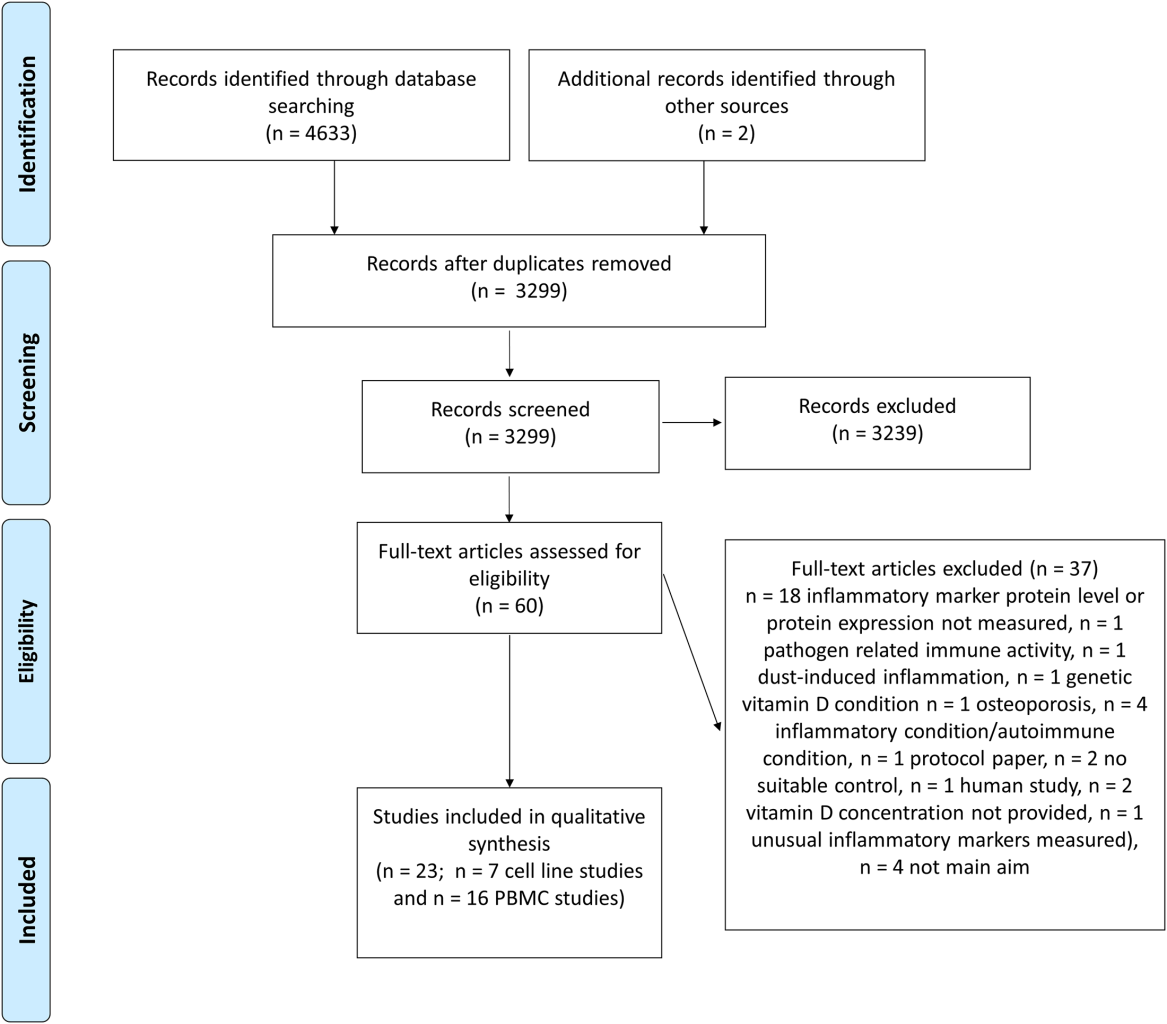
PRISMA flow diagram depicting the systematic study selection process. PBMC, peripheral blood mononuclear cells.

A literature search was conducted independently by two reviewers (EKC and KNK) using Pubmed, Science Direct, Scopus, Wiley and Medline (search updated 19^th^ June 2015). A third independent reviewer was consulted to resolve discrepancies (PN). The search strategy used the following search terms: Vitamin D or cholecalciferol or 1,25-dihydroxyvitamin or 25-hydroxy-Vitamin D and Inflam* or cytokine* and supplement* or cell*. These terms were searched in the abstract, title or keywords. Inclusion criteria for study selection included articles reported in the English language and within the time period of 2000 to 2015. The study selection process was mapped according to the Preferred Reporting Items for Systematic Reviews and Meta-Analyses guidelines (PRISMA) and can be seen in Fig 1. Study characteristics such as cell line, cell type, participant demographics, vitamin D form, dose, duration of exposure, presence of an inflammatory stimulus, direction of inflammatory marker change and pathway were extracted by two independent reviewers (EKC and KNK) and cross-checked as required (PN) (S2 Table. PRISMA Checklist).

## Results

### Cell line studies

**Fig 2:**
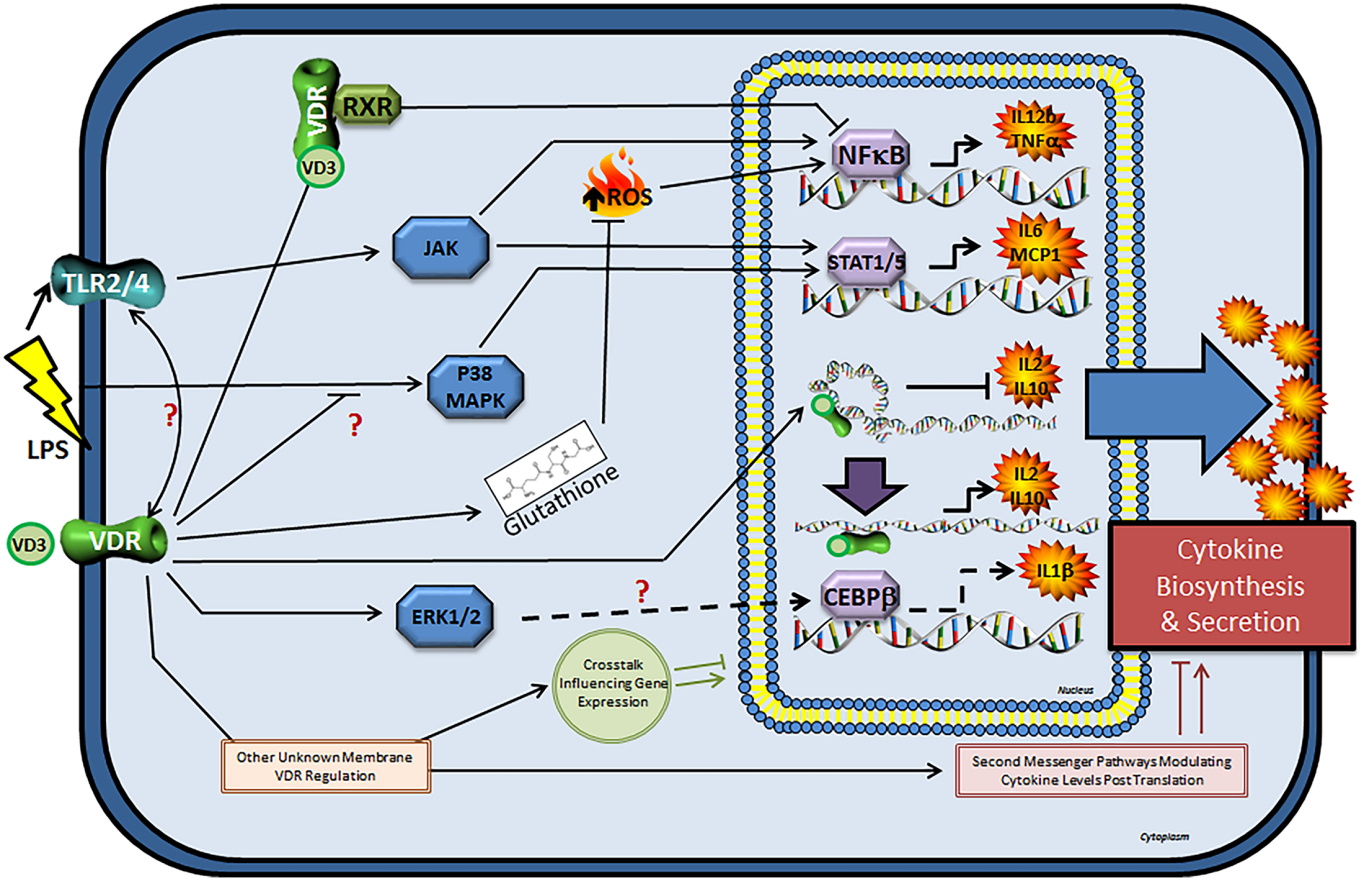
Overview of VDR-mediated regulation of cytokine transcription, production and secretion in immune cells. Interaction of VD3 and VDR leads to anti-inflammatory effects through negative regulation of NFκB and STAT1/5-mediated signalling. This results in decreased transcription of TNF-α, IL-6, MCP1 and IL-12β. VDR activation promotes increased intracellular glutathione levels that partially or fully attenuates excessive ROS production (ROS can activate pro-inflammatory NFκB signalling). Activated VDR regulates transcription of IL-2 and IL-10 through epigenetic and conformational changes in the promoter region of these genes. VDR association with the promoter region occurs in a cyclic fashion, which leads to initial gene suppression, followed by upregulation of IL-2 and IL-10 expression after 48 hours. Pro-inflammatory effects of VD3 were reported and suggested to be linked to increased IL-1β production possibly related to increased ERK1/2 phosphorylation and the transcription factor CEBPβ. The VDR is believed to modulate pro-inflammatory TLR expression both positively and negatively, but the mechanisms are unknown. Plasma membrane associated VDR may induce rapid effects through non-genomic pathways such as modulation of intracellular calcium levels, parathyroid hormone G-protein coupled or other second messenger systems. Non-genomic pathways may cooperate with genomic pathways to influence gene expression. CCAAT/enhancer binding protein beta (CEBPβ), extracellular signal-regulated kinase1/2 (ERK1/2), janus kinase (JAK), monocyte chemotatic protein1 (MCP-1), nuclear factor kappa light chain enhancer of activated B cells (NFκB), mitogen activated protein kinase (p38 MAPK), retinoid X receptor (RXR), reactive oxygen species (ROS), signal transducer and activator of transcription1/5 (STAT1/5), toll-like receptor-2/4 (TLR2/4), tumour necrosis factor alpha (TNF-α), vitamin D3 (VD3), vitamin D receptor (VDR).

Seven cell line studies (Table 1) were identified. Six out of the seven studies used the THP-1 cell line, while two studies used the U937 cell line and one study used Jurkat cells. All cell line studies administered vitamin D in the form of 1,25(OH)_2_ and one study also used 25(OH)D. All studies except one administered vitamin D in conjunction with an inflammatory stimulus. Overall, the majority of cell line studies (4 out of 7) reported that vitamin D had an anti-inflammatory effect, one study reported mixed effects and two studies reported a pro-inflammatory effect. The most common concentration of 1,25(OH)_2_ that indicated an anti-inflammatory effect was 10 nM (4 out of 7 studies). Mechanisms likely to mediate the anti-inflammatory effect of vitamin D included suppressed phosphorylated p38 (pp38) expression [40], reduced expression of p-STAT5 [41], and decreased reactive oxygen species levels due to increased cellular glutathione [42] (Fig 2).

**Table 1:** Cell studies examining the impact of vitamin D on inflammation.

Study	Cell line/cell type	Vitamin D form, dose and time	Stimulation method	Significant inflammatory marker change	Net effect
Cell line studies					
Tulk et al 2015 [47]	THP-1	1,25(OH)_2_ (0, 0.1, 10, 100, 500 nM) 25(OH)D (0, 0.1, 10, 100, 500 nM)	PMA 100 nM overnight	IL-1β ↑	Pro-inflammatory (≥100 nM 25(OH)D and ≥1 nM 1,25(OH)_2_D)
Wang et al 2014 [40]	THP-1	1,25(OH)_2_ (0, 100, 1000, 10000 nM) for 2 h	LPS 0.2 ug/ml for 6, 24 and 48 h	MCP-1 ↓	Anti-inflammatory
Yang et al 2012 [41]	THP-1	1,25(OH)_2_ (0, 100 nM) for 48 h	LPS 1 ug/ml + IL-15 100 ng/ml for 4 h	IL-6, MCP-1 ↓	Anti-inflammatory
Matilainen et al 2010 [48]	THP-1	1,25(OH)2 (0, 10 nM) for 48 h	LPS 100 ng/ml for 24 h	IL-10 mRNA ↓ (8 h) then ↑ (48 h)	Anti-inflammatory
Matilainen et al 2010 [49]	THP1 + Jurkat lymphocyte cells	1,25(OH)_2_ (0, 10 nM) for 24 h	LPS 100 ng/ml for 24 h or 2 ug/ml PHA and 50 ng/ml TPA	IL-2, IL-10 mRNA ↓ (3, 6 h) then ↑(24 h) IL-12 mRNA ↓ (6h)	Mixed
Lee et al 2011 [50]	U937 THP	1,25(OH)_2_ (0, 10 nM) for 24 h	PMA	IL-1β protein expression and protein level ↑	Pro-inflammatory
Jain & Micinski 2013 [42]	U937 monocytes	1,25(OH)_2_ (0, 10, 25 nM) for 24h	No inflammatory stimulant	IL-8, MCP-1 ↓	Anti-inflammatory
PBMC studies					
Cantorna 2015^a^ [51]	PBMCs	1,25(OH)_2_ (0, 10, 50 nM) for 72 hours	α-Galactoceramide for 72 hours	INF- γ ↓ IL-4 ↑	Anti-inflammatory
Ojaimi 2013^b^ et al [52]	PBMCs	Cholecalciferol, 50,000 IU daily for 10 days, then 50000 monthly for 3 months	Pam3Cys 100 ng/ml PolyI:C 10 μg/ml LPS 100 ng/ml or unstimulated media for 24 h.	TNF-α, IL-6 ↓, then NC Unstimulated showed no effect as basal cytokine production was so low	Anti-inflammatory (when serum levels >100 nM)
Khoo 2011 et al [36]	PBMCs	1,25(OH)_2_ 0 or 10^-7^ M (100 nM) for 30 min	Pam3Cys 10 mg/ml or LPS 10 ng/ml or RPMI control for 24 h	IL-6, TNF-α ↓	Anti-inflammatory
Rausch-Fan et al 2002 [44]	PBMCs	1,25(OH)_2_ (0.01 to 100 nM) for 48 h	PMA 10 ng/ml and ionomycin 1.25 uM	INF- γ, IL-2, IL-10, TNF-α, IL-12, IL-1β ↓, IL-5, IL-10 ↑, IL-4 NC	Anti-inflammatory (10^-8^, 10^-7^ M)
Takahashi 2002 [53]	PBMCs	1,25(OH)_2_ (0, 0.1, 100nM) for 2 h, 4 h, 8 h and 24 h	LPS 1 ug/ml or IL-1β 10ng/ml	IL-8 ↓ (24 h)	Anti-inflammatory
Giovanni 2001 et al [45]	PBMCs	1,25(OH)_2_ (25, 50, 100 ng) for 12 h	LPS 100 ng/ml	TNF-α, IL-1β, IL-6, IL-10 ↓, dose-dependent NE when PBMC incubated without LPS	Anti-inflammatory
Di Rosa 2012 et al [46]	Monocyte derived macrophages & monocytes	1,25(OH)_2_ (0, 1000 nM) for 24 h	alone or in combination with TNF-α 100 U/ml or LPS 50 ng/ml for 2 h	Monocytes: IL-1β, IL-6, TNF-α mRNA NC Macrophages + LPS: IL-1β, IL-6 mNRA NC TNF-α mRNA ↑, Macrophages + TNF- α: IL-1β mNRA NC IL-6, TNF-α mRNA ↓, Macrophages without stimulation: IL-1β, IL-6, TNF-α ↓	Monocytes: No effect; Macrophages: Anti-inflammatory
Zhang 2012 et al [22]	Monocytes	1,25(OH)_2_ (0, 1, 10 nM) for 24 h25(OH)D (0, 15 ng/ml, 30 ng/ml, 50 ng/ml and 70 ng/ml) for 24 h	10 ng/ml LPS for 24 h	IL-6 ↓ dose-response	Anti-inflammatory
Du 2009 [54]	Monocytes	1,25(OH)_2_ (0, 100 nM) for 48 h	LPS 100 ng/ml and LTA 10 ug/ml for 3 h	TNF- α, IL1β ↓	Anti-inflammatory
Sadeghi 2006 et al [43]	Monocytes	1,25(OH)_2_ (0.01 to 100 nM) for 48 h	10 ng LPS or 10 ug LTA for 4 h	TNF- α ↓, dose-response	Anti-inflammatory (10^-9^ to 10^-7^ M)
Sloka 2011 et al [32]	T cells	1,25(OH)_2_ (0, 0.1 and 10 nM) of 1,25(OH)_2_	mouse anti-human CD3 10 or 1000 ng/mL for 3 days	IFN- γ, IL-17 ↓, IL-5 ↑	Anti-inflammatory
Thien 2005 et al [55]	T cells	1,25(OH)_2_ (0, 10 nM) for 7–14 days	IL-4 500 U/mL or IL-12 200 U/mL	INF- γ, IL-4, IL-6, IL-13 ↑, IL-2 ↓	Mixed
Khoo 2011 et al [36]	Treg cells, T convential cells	1,25(OH)_2_ (0, 100 nM) for 8 days	Treg and Tconv cells were stimulated with anti-CD3/anti-CD28 monoclonal antibody-coated microbeads and PMA	IL-4, IL-10 ↑, TNF-α ↑, IL-2, IFN-γ, IL-17 NC	Mixed
Zhang, Leung & Goleva 2013 [56]	PBMCs-CD14^+^ and CD14^-^ T cells	1,25(OH)_2_ (0, 10 nM) for 24 h	LPS 10ng/ml for 6 h	IL-6 ↓	Anti-inflammatory
Jeffery 2009 et al ^a^ [57]	T cells CD4^+^CD25^-^	1,25(OH)_2_ (0, 100 nM) for 5 days	anti-CD3- and anti-CD28 Antibody-coated beads	IFN- γ, IL-2, IL-17, IL-21 ↓, IL-10 ↑	Anti-inflammatory
Jirapongsananuruk 2000 et al [58]	PBMCs-lymphocyte	1,25(OH)_2_ (0, 1000 nM) for 72 h	anti-CD3	IL-5, IL-13 ↑ IFN- γ ↓	Anti-inflammatory

25-hydroxyvitamin D (25(OH)D), 1,25-dihydroxyvitamin D (1,25(OH)_2_), interferon gamma IFN-γ, interleukin 1β (IL1β), interleukin 2 (IL-2), interleukin 4 (IL-4), interleukin 5 (IL-5), interleukin 6 (IL-6), interleukin 8 (IL-8), interleukin 10 (IL-10), interleukin 12 (IL-12), interleukin 13 (IL-13), interleukin 15 (IL-15), interleukin 17 (IL-17), interleukin 21 (IL-21), monocyte chemotatic protein-1 (MCP-1), no change NC, peripheral blood mononuclear cells (PBMCs), tumor necrosis factor alpha (TNF- α).

^a^ Health status of participants unknown

^b^ Study conducted in participants with inadequate vitamin D status (serum 25(OH)D < 50 nM

### PBMC studies

We identified sixteen studies that used PBMCs (Table 1). Of these, fifteen studies administered vitamin D in the form of 1,25(OH)_2_, one study used cholecalciferol and one study also used 25(OH)D. All studies examined the effect of vitamin D in conjunction with an inflammatory stimulus. Of these, three studies also examined the effect of vitamin D alone without an inflammatory stimulus. The majority of PBMC studies showed that vitamin D had an anti-inflammatory effect (14 out of 16 studies), with two studies reporting mixed effects (Table 1). PBMCs were obtained from healthy participants in fourteen out of sixteen studies and the health status of participants in two studies was unknown. Six studies used PBMCs, four studies used monocytes, one study used macrophages, five studies used T-cells and one study used mixed lymphocytes. The two most common concentrations of 1,25(OH)_2_ that elicited an anti-inflammatory response was 10 nM (7 studies) and 100 nM (7 studies). Four studies demonstrated a dose-dependent response of vitamin D with respect to reducing inflammation, with 1 nM and 10 nM concentrations causing the greatest effects [22, 43–45]. Mechanisms likely to mediate the anti-inflammatory effect of vitamin D included decreased protein expression of toll-like receptor-2 (TLR-2) [43, 46] and toll-like receptor-4 (TLR-4) [43, 46], elevated trans-acting T-cell-specific transcription factor (GATA-3) mRNA through elevating upstream factor signal transducer and activator of transcription 6 (STAT6) [32], VDR [43], lower levels of pp38 and p42/42 (ERK1/2) [22, 43], and localization of p65 [43] (Fig 2).

## Discussion

Inadequate vitamin D status is commonly observed in populations across the world [12]. This observation parallels the high prevalence of obesity-related chronic diseases that carry a heavy inflammatory burden. Our objective was to comprehensively review the cellular evidence linking vitamin D with the inflammatory profile of human-derived immune cells.

Our results demonstrated that the active form of vitamin D decreased the inflammatory status of cellular models. We found evidence that vitamin D was able to indirectly quench ROS, which are accepted as a major factor in the onset and development of chronic diseases including type 2 diabetes [59]. We also found evidence that vitamin D decreased TLR expression, which is increased in both immune cells and adipose tissue from overweight and obese subjects [60]. Furthermore, TLR activation has been implicated in mechanisms of obesity-related insulin resistance and metabolic dysfunction [61, 62]. TLRs are shown to be stimulated by both endogenous and exogenous factors such as dietary saturated fatty acids [63] and resistin [64], both of these factors induce inflammatory changes in circulating immune cells [65]. The TLR transmembrane proteins subsequently initiate classical signaling cascades leading to the activation of transcription factors, such as NFκB [66] and cytokine production [62]. TLR pathways also stimulate a variety of cellular responses including host defense in response to microbial products, and subsequently impact energy metabolism. Stimulated NFκB exerts its action through binding to DNA and inducing the transcription of many genes involved in various aspects of innate and adaptive immune responses, such as those coding for cytokines, growth factors, adhesion molecules [67], and multiple genes that regulate cellular differentiation, survival and proliferation [68]. Clearly, evidence suggests that 1,25(OH)_2_ acts to suppress NFκB activity. It is possible that vitamin D acts through suppression of the NFκB transcriptional activity, or through regulation of cellular ROS levels, which subsequently alter NFκB transcriptional activity. However, the precise pathway(s) awaits confirmation. It is also possible, that inhibition of inflammatory signalling by vitamin D could happen upstream of modulation of transcriptional factor action.

It is also possible that vitamin D may be exerting anti-inflammatory effects through non-genomic pathways initiated at the plasma membrane VDR [35] (Fig 2). Binding of 1,25(OH)2D at the plasma membrane VDR may result in the activation of one or more second messenger systems, such as phospholipase C (and subsequently protein kinase C, through generation of both diacylglycerol and a rise in intracellular Ca^2+^), and G protein-coupled receptors. Furthermore, non-genomic pathways could cooperate with the classical genomic pathway via cross-talk to influence gene expression. Perhaps application of a systems biology approach may reveal additional mechanisms of action.

We are unable to comment on whether 25(OH)D modulates inflammation, as few studies used this form of vitamin D. However, we and others [22] believe that prevailing 25(OH)D levels may be crucial since they influence local tissue concentrations of the active vitamin D metabolite [69]. Serum 25(OH)D levels as high as 120 nmol/L may be necessary for optimal immune function [52]. Indeed, it was reported [52] that the anti-inflammatory benefit of vitamin D was only seen in those individuals in whom 25(OH)D rose to >100 nmol/L. Beneficial effects disappeared when vitamin D status dropped to below 100 nmol/L. Since human recommendations for good health are based on appropriate serum levels of 25(OH)D, cellular studies could asses the effect of various doses of 25(OH)D that reflect/mimic whole body circulating concentrations of the hormone. In this systematic review, we did not investigate the impact of vitamin D on subsequent cell function. Potential therapeutic agents like vitamin D which target immune pathways such as NFκB, ROS quenching and JAK, must be able to antagonize the harmful effects of inflammation without affecting host defense functions. Further studies are therefore required to determine the full effect of vitamin D on other parameters of immune and cellular function.

## Conclusion

Vitamin D consistently displayed anti-inflammatory effects in both human cell lines and PBMCs. Cellular studies which examine the impact of 25(OH)D on inflammatory status and responses are now required. Clinical studies are warranted to confirm whether supplementation and elevation in circulating vitamin D levels are able to modulate inflammation and improve outcomes or prevent chronic disease.

## Supporting Information

S1 Table Systematic review protocol. (PDF)

S2 Table PRISMA checklist. (PDF)

## Abbreviations

(CRP): C-reactive protein
(IL-1β): interleukin-1β
(IL-2): interleukin-2
(IL-4): interleukin-4
(IL-6): interleukin-6
(IL-8): interleukin-8
(IL-10): interleukin-10
(IL-12): interleukin-12
(IL-23): interleukin-23
(MCP-1): monocyte chemotactic protein 1
(nVDR): nuclear vitamin D receptor
(PBMCs): peripheral blood mononuclear cells
(PRISMA): preferred reporting items for systematic reviews and meta-analyses
(TNF-α): toll-like receptor (TLR), tumour necrosis factor α
(VDR): vitamin D receptor
