# Supplementary material for: The Impact of Vitamin D Levels on Inflammatory Status: A Systematic Review of Immune Cell Studies
Source: PLoS One. 2015 Nov 3;10(11):e0141770. doi: 10.1371/journal.pone.0141770 (PMC4631349; doi:10.1371/journal.pone.0141770)
Supplement: S1 Table — (PDF) [file pone.0141770.s001.pdf]

PROSPERO International prospective register of systematic reviews

Review title and timescale

1 Review title

Give the working title of the review. This must be in English. Ideally it should state succinctly the interventions or exposures being reviewed and the associated health or social problem being addressed in the review.

The effect of vitamin D manipulation on the inflammatory profile: a systematic review of immune cell studies

2 Original language title

For reviews in languages other than English, this field should be used to enter the title in the language of the review. This will be displayed together with the English language title.

3 Anticipated or actual start date

Give the date when the systematic review commenced, or is expected to commence.

02/02/2015

4 Anticipated completion date

Give the date by which the review is expected to be completed.

02/07/2015

5 Stage of review at time of this submission

Indicate the stage of progress of the review by ticking the relevant boxes. Reviews that have progressed beyond the point of completing data extraction at the time of initial registration are not eligible for inclusion in PROSPERO. This field should be updated when any amendments are made to a published record.

The review has not yet started ✕

Review stage	Started	Completed
Preliminary searches	Yes	No
Piloting of the study selection process	Yes	No
Formal screening of search results against eligibility criteria	Yes	No
Data extraction	No	No
Risk of bias (quality) assessment	No	No
Data analysis	No	No

Provide any other relevant information about the stage of the review here.

Review team details

6 Named contact

The named contact acts as the guarantor for the accuracy of the information presented in the register record.

Mario Soares

7 Named contact email

Enter the electronic mail address of the named contact.

m.soares@curtin.edu.au

8 Named contact address

Enter the full postal address for the named contact.

School of Public Health Curtin University Bentley Campus, Kent Street, Perth, Western Australia 6845

9 Named contact phone number

Enter the telephone number for the named contact, including international dialing code.

+ 61-8- 92663220

10 Organisational affiliation of the review

Full title of the organisational affiliations for this review, and website address if available. This field may be completed as 'None' if the review is not affiliated to any organisation.

Curtin University

Website address:

www.curtin.edu.au

11 Review team members and their organisational affiliations

Give the title, first name and last name of all members of the team working directly on the review. Give the organisational affiliations of each member of the review team.

Title	First name	Last name	Affiliation
Dr	Mario	Soares	Curtin University
Miss	Emily	Calton	Curtin University
Dr	Kevin	Keane	Curtin University
Professor	Philip	Newsholme	Curtin University

12 Funding sources/sponsors

Give details of the individuals, organizations, groups or other legal entities who take responsibility for initiating, managing, sponsoring and/or financing the review. Any unique identification numbers assigned to the review by the individuals or bodies listed should be included.

School of Public Health and School of Biomedical Sciences, Curtin University. EC is a recipient of an APA scholarship provided by the Australian Government.

13 Conflicts of interest

List any conditions that could lead to actual or perceived undue influence on judgements concerning the main topic investigated in the review.

Are there any actual or potential conflicts of interest?

None known

14 Collaborators

Give the name, affiliation and role of any individuals or organisations who are working on the review but who are not listed as review team members.

Title First name Last name Organisation details

Review methods

15 Review question(s)

State the question(s) to be addressed / review objectives. Please complete a separate box for each question.

What is the impact of vitamin D manipulation on immune cell lines derived from humans and peripheral blood mononuclear cells obtained directly from humans on inflammatory markers/anti-inflammatory markers

16 Searches

Give details of the sources to be searched, and any restrictions (e.g. language or publication period). The full search strategy is not required, but may be supplied as a link or attachment.

PubMed, Scopus, Science Direct, MEDLINE, and Web of Science will be searched. Abstract, title and keywords to be searched Restricted to studies published within 2000-2015 and reported in the English language

17 URL to search strategy

If you have one, give the link to your search strategy here. Alternatively you can e-mail this to PROSPERO and we will store and link to it.

I give permission for this file to be made publicly available

Yes

18 Condition or domain being studied

Give a short description of the disease, condition or healthcare domain being studied. This could include health and wellbeing outcomes.

Change in inflammatory/anti-inflammatory markers

19 Participants/population

Give summary criteria for the participants or populations being studied by the review. The preferred format includes details of both inclusion and exclusion criteria.

Inclusion criteria: immune cells originating from humans (either cell lines or obtained from blood) The peripheral blood mononuclear cells must be from healthy participants or those with obesity-related chronic inflammatory disorders The peripheral blood mononuclear cells must be from adults Exclusion criteria: participants have a highly active inflammatory condition that is not associated with obesity or chronic disease peripheral blood mononuclear cells come from children or babies

20 Intervention(s), exposure(s)

Give full and clear descriptions of the nature of the interventions or the exposures to be reviewed

Inclusion criteria: cells are exposed to a form of vitamin D there is a control (no vitamin D) vitamin D is given alone or in conjunction with a stimulatory agent and not given in conjunction with a drug common inflammatory markers are measured (e.g. cytokines, adiponectin) protein level, protein expression or functional tests of cytokines are reported primary aim of the study is to examine the effect of vitamin D on inflammatory markers Exclusion cells are not exposed to vitamin d vitamin D is the control group vitamin D is given in combination with a drug neither protein level or protein expression or gene expression with functional/mechanistic results are measured the impact of vitamin D on inflammatory markers is not the main aim of the study

21 Comparator(s)/control

Where relevant, give details of the alternatives against which the main subject/topic of the review will be compared (e.g. another intervention or a non-exposed control group).

Non-exposed control group (no vitamin D) Inclusion: 0 vitamin D is the control group Exclusion: studies which use vitamin D as the control group or another form of vitamin D

22 Types of study to be included initially

Give details of the study designs to be included in the review. If there are no restrictions on the types of study design eligible for inclusion, this should be stated.

Inclusion criteria: cell studies which are directly exposed to a form of vitamin D there is a control (no vitamin D) Exclusion criteria study design is not a cellular study

23 Context

Give summary details of the setting and other relevant characteristics which help define the inclusion or exclusion criteria.

24 Primary outcome(s)

Give the most important outcomes.

The impact of vitamin D compared to no vitamin D on inflammatory markers in immune cell lines and immune cells obtained from peripheral blood mononuclear cells

Give information on timing and effect measures, as appropriate.

25 Secondary outcomes

List any additional outcomes that will be addressed. If there are no secondary outcomes enter None.

What are the mechanisms by which vitamin D exerts its effect on inflammatory markers?

Give information on timing and effect measures, as appropriate.

26 Data extraction, (selection and coding)

Give the procedure for selecting studies for the review and extracting data, including the number of researchers involved and how discrepancies will be resolved. List the data to be extracted.

Literature search will be conducted using Pubmed, Science Direct, Scopus, Web of Science and MEDLINE. Restricted to studies in English and from 2000-2015. Duplicates removed. Study inclusion will be assessed by two independent reviewers and a third independent reviewer will be consulted if discrepancy exists. Data extraction will occur for cell type, vitamin D form, dose and duration of exposure, presence or absence of an inflammatory stimulus, inflammatory marker change and mechanisms of action by two independent reviewers and cross-checked by a third independent reviewer when discrepancy exists.

27 **Risk of bias (quality) assessment**
State whether and how risk of bias will be assessed, how the quality of individual studies will be assessed, and whether and how this will influence the planned synthesis.
The quality of each study will be assessed as good (and the study therefore included) if the study provides vital information including the cell type studied, vitamin D form, vitamin D concentration, inflammatory marker change and if the results and discussion are consistent

28 **Strategy for data synthesis**
Give the planned general approach to be used, for example whether the data to be used will be aggregate or at the level of individual participants, and whether a quantitative or narrative (descriptive) synthesis is planned. Where appropriate a brief outline of analytic approach should be given.
A semi-quantitative descriptive synthesis is planned whereby the number of studies which showed an anti-inflammatory or pro-inflammatory effect or no effect will be described.

29 **Analysis of subgroups or subsets**
Give any planned exploration of subgroups or subsets within the review. 'None planned' is a valid response if no subgroup analyses are planned.
If there are enough studies in more than 1 cell line type (at least 3 studies in each type), results will be divided according to type of cell line. Studies will be discussed based on the form of vitamin D administered. Studies will be divided based on whether they use vitamin D alone or stimulate the cells with an inflammatory agent if there are enough studies in each situation (at least 3 studies with and without an inflammatory stimulus).

Review general information

30 **Type of review**
Select the type of review from the drop down list.
Intervention

31 **Language**
Select the language(s) in which the review is being written and will be made available, from the drop down list. Use the control key to select more than one language.
English
Will a summary/abstract be made available in English?
Yes

32 **Country**
Select the country in which the review is being carried out from the drop down list. For multi-national collaborations select all the countries involved. Use the control key to select more than one country.
Australia

33 **Other registration details**
Give the name of any organisation where the systematic review title or protocol is registered together with any unique identification number assigned. If extracted data will be stored and made available through a repository such as the Systematic Review Data Repository (SRDR), details and a link should be included here.

34 **Reference and/or URL for published protocol**
Give the citation for the published protocol, if there is one.
Give the link to the published protocol, if there is one. This may be to an external site or to a protocol deposited with CRD in pdf format.

I give permission for this file to be made publicly available
Yes

35 **Dissemination plans**
Give brief details of plans for communicating essential messages from the review to the appropriate audiences.
publication and thesis
Do you intend to publish the review on completion?
Yes

36 **Keywords**
Give words or phrases that best describe the review. (One word per box, create a new box for each term)
systematic review
cell
inflammation
cytokine
vitamin D
immune

37 **Details of any existing review of the same topic by the same authors**
Give details of earlier versions of the systematic review if an update of an existing review is being registered, including full bibliographic reference if possible.

38 **Current review status**
Review status should be updated when the review is completed and when it is published.
Ongoing

39 **Any additional information**
Provide any further information the review team consider relevant to the registration of the review.

40 **Details of final report/publication(s)**
This field should be left empty until details of the completed review are available.
Give the full citation for the final report or publication of the systematic review.
Give the URL where available.
